# Understanding barriers to medication, dietary, and lifestyle treatments prescribed in polycystic kidney disease

**DOI:** 10.1186/s12882-017-0641-3

**Published:** 2017-07-05

**Authors:** Wen-Ching Tran, David Huynh, Tea Chan, Catherine A. Chesla, Meyeon Park

**Affiliations:** 1 School of Medicine, University of California, San Francisco, CA USA; 2 Division of Nephrology, Department of Medicine, University of California, 521 Parnassus Ave, C443, Box 0532, San Francisco, CA 94143 USA; 3 Department of Family Health Care Nursing, University of California, San Francisco, CA USA

**Keywords:** Polycystic kidney disease, Illness perception models, Treatment barriers, Lifestyle

## Abstract

**Background:**

Autosomal dominant polycystic kidney disease (PKD) is the most common genetic renal disease and the fourth leading cause of end-stage renal disease in the United States. Although there is no cure for PKD, several treatments are considered to be beneficial, including blood pressure control, exercise, low-salt diet, and high volume water intake. However, levels of understanding of the importance of these treatments and adherence to these recommendations vary among patients. This study explores illness perception models of patients with PKD to reveal barriers in adherence to prescribed therapies; satisfaction with medical care; and sources of medical information.

**Methods:**

We designed a phenomenological interview study to evaluate illness perception models of individuals with PKD. Patients were identified from the national PKD Foundation e-mail distribution list (*N* = 190) and responded voluntarily to an introductory survey (*N* = 50). Seventeen PKD patients in the Bay Area were scheduled for one-on-one in-depth interviews with one trained interviewer (W-CT). Open-ended questions administered with an interview guide were used to evaluate patients’ beliefs.

**Results:**

Mean age was 56.6 +/− 12 years (range 29–78); 65% were female. Many of the PKD patients in this study were highly motivated and willing to incorporate blood pressure, exercise, low-salt diet, and high volume water intake into their daily routines. Barriers to adherence to these therapies include personal beliefs and confusion due to unclear recommendations.

**Conclusions:**

These findings suggest there is variability between what patients understand about their disease and treatments and what they believe their doctors have told them. Not all physicians focus on lifestyle-based treatments, but the majority of PKD patients in our study are motivated and willing to incorporate blood pressure control, exercise, low-salt diet, and high volume water intake into their daily routines and would like specific recommendations on how to implement these. These findings support a role for further exploring patient beliefs about the disease and its necessary treatments in order to design strategies to improve communication and meet the needs of these patients.

**Electronic supplementary material:**

The online version of this article (doi:10.1186/s12882-017-0641-3) contains supplementary material, which is available to authorized users.

## Background

Autosomal dominant polycystic kidney disease (PKD) is the most common genetic renal disease and the fourth leading cause of end-stage kidney disease in the United States [1]. Individuals with PKD have a high risk of hypertension and cardiovascular disease, independent of renal function [2]. Currently, there is no cure for PKD. Consequently, many individuals experience a high degree of emotional distress from this disease, and uncertainty about disease progression is a major source of distress [3]. The inability to influence disease progression is a major source of feelings of helplessness and hopelessness in these patients [3].

Despite the absence of a cure, several recommended treatments may attenuate progression of kidney disease and / or reduce risk of cardiovascular events. These include 1) blood pressure control [4]; 2) exercise [5]; 3) low-salt diet [6]; and 4) high volume water intake [7]. The recently published HALT-PKD trial [4] demonstrated that in individuals with preserved renal function, using an angiotensin converting enzyme inhibitor (ACE-I) to a target systolic blood pressure of less than 110 mmHg is beneficial for several meaningful renal and cardiovascular outcomes [4]. Adherence to blood pressure medication is notoriously low [8], and to our knowledge, adherence specifically in the PKD population has not been studied. Additionally, while aerobic exercise has been shown to be beneficial in the general population with hypertension [5], it is uncertain how many PKD patients perform the daily physical activity recommended for the general adult population [9]. As PKD can cause major pain and discomfort due to enlargement of kidneys and hemorrhage of cysts, some patients actually avoid exercise, either on recommendation of a physician or based on their own experience. Although the optimal target is controversial, a low-salt diet has been shown to be of clear benefit for individuals with hypertension [6]. The extent of patient counseling and adherence to a low-salt diet is unknown in the PKD community. Finally, a suppression of the antidiuretic hormone (ADH) with high volume water intake is a known method to attenuate progression of kidney cyst growth [10, 11], and the medication tolvaptan (an ADH antagonist) is currently an approved therapy for PKD in Japan, Canada, and Europe. Although many individuals are told to “drink a lot of water” to achieve this effect [7], adherence with this prescription is uncertain.

Levels of understanding of the importance of these treatments and adherence to these recommendations may vary among patients. Illness models have been used in the study of hypertension and other chronic diseases to study barriers to adherence [12]. The study of illness perception models has revealed that health-related behavior is strongly influenced by ideas around five components, or illness representations. These include cause, nature (including seriousness), and treatment of the disease (including hoped for outcomes from those treatments) [13, 14]. An understanding of these beliefs is the first step toward designing meaningful interventions to improve adherence to treatment. This study explores illness perception models, focusing most centrally on patients perceptions of treatments needed among patients with PKD to reveal barriers to adherence to prescribed therapies; satisfaction with medical care; and sources of medical information.

## Methods

We designed a phenomenological interview study to evaluate illness perception models of individuals with PKD [15]. Patients were initially identified from the national PKD Foundation e-mail distribution list (*N* = 190) and voluntarily responded to an introductory survey (*N* = 50). Seventeen PKD patients in the Bay Area came to UCSF for one-on-one in-depth interviews with a trained interviewer (W-CT). The characteristics of patients who responded to the survey and the interview participants are summarized in Table 1. The PKD Foundation does not uniformly collect demographic information on members of its e-mail distribution list and thus information on the individuals who received our survey is not available. The study was approved by the UCSF IRB and all participants gave written informed consent.

**Table 1:** Demographics

	Survey respondents (n= 50)	Interview participants (n= 17)
Mean Age	55.5 +/− 13.8 years (Range = 25–89)	56.6 +/− 12 years (Range = 29–78)
Gender		
Female	26 (52%)	11 (64.7%)
Male	21 (42%)	6 (35.3%)
Did not respond	3 (6%)	0
Race/Ethnicity		
White, Hispanic or Latino	7 (14%)	3 (17.6%)
White, not Hispanic or Latino	31 (62%)	11 (64.7%)
Black	5 (10%)	1 (5.9%)
Asian or Pacific Islander	4 (8%)	1 (5.9%)
Native American	1 (2%)	0
Prefer not to answer	1 (2%)	1 (5.9%)
Did not respond	1 (2%)	0
Currently on dialysis	3 (6%)	0
Post-kidney transplant	4 (8%)	4 (23.5%)

Interviews were conducted in a private classroom. Open-ended questions administered with an interview guide were used to evaluate patient beliefs on 1) the treatments available for PKD; 2) whether they had been prescribed blood pressure control, exercise, low-salt diet, and high volume water intake and their adherence to these treatments; and 3) sources of medical information. The interview guide was developed based on previous studies conducted by the authors in different patient populations [16, 17], as well as on personal experience with the current patient population using guidance from Kvale [18]. The interview guide followed the elements of the illness models as recommended by Kleinman [14], but particular attention was placed on participants beliefs and practices regarding four established recommendations for lifestyle change in PKD. The interview guide was drafted and reviewed by all authors and revised accordingly. Pilot testing could not be performed due to limited budget, time for completion of the project, and patient availability. The full interview guide is provided in Additional file 1. The sample size was determined by budget, as patients were remunerated for their time and participation with a token gift card and reimbursement for parking / transportation costs.

Each interview was approximately one hour long. The interviews were audio-recorded without identifiers using the Voice Recorder app on an iPad. These recordings were transcribed using rev.com. Transcriptions were reviewed for quality, and the responses were initially organized by question on an Excel spreadsheet. The responses were then coded independently by two trained individuals (W-CT and DH). Discrepancies were discussed until consensus was established. The coded data was grouped into themes [19]. The general framework for analysis was to note participant’s beliefs about treatments needed, and how these related to PKD self-care practices. Additionally, we explored how patients came to believe certain treatments were efficacious, despite what they had been told. We did not have an a priori framework for themes. Software was not used to track coding or facilitate analysis due to budgetary limitations. No repeat interviews were done for the same reason. Transcripts and findings were not shared with participants for comments and/or corrections.

## Results

### Demographics

Mean age of participants was 56.6 +/− 12 years (range 29–78). Sixty-five percent were female. The majority were non-Hispanic/Latino Caucasians. None of the participants were receiving dialysis, while four were post-kidney transplant (Table 1). Many of the PKD patients in this study were highly motivated and willing to incorporate blood pressure, exercise, low-salt diet, and high volume water intake into their daily routines. Barriers to adherence to these therapies include personal beliefs and confusion due to limited recommendations.

### Theme 1: Patients motivated by treatment beliefs

Sixteen of the 17 participants reported believing that blood pressure affects PKD and were also prescribed blood pressure medication. Of the 16 participants who were prescribed blood pressure medication, 12 (75%) reported taking his or her medication everyday (Table 2). Patients felt they “have to” and “need to” take their medication every day because “there’s so little that you can control.” Many participants take extra steps, such as using pill boxes, leaving the medication at a visible location, or having another person provide reminders, to make sure they take their medications daily.

**Table 2:** Summary of Results

Treatment	Belief	*N*	Recommendations	*N*	Adherence	*N*	Challenges to Adherence
Blood pressure	Believes blood pressure affects PKD	16	Prescribed blood pressure medication	16	Number of days per week when remember to take medication:		• Believes diet and exercise are better than drugs • Forgets when feeling well • Forgets when rushed, busy, or tired
					<7 days/week	4	
					7 days/week	12	
Exercise	Believes exercise or physical activity affects PKD	14	Told to avoid certain types of exercise or physical activity	11	Does not adhere to recommendations to avoid certain exercises of physical activity	2	• Activity helps mentally • Disagrees with recommendations (that exercise is harmful)
Diet	Believes diet affects PKD	16	Told to follow certain diets	15	Adherence to low-salt diet:		• Absence of or conflicting recommendations • Affects social aspects of eating • Difficult to measure
			• By non-physician sources	10	Does not add salt to food	12	
					Avoids salty food	4	
					Does not eat out/ Cooks own meals	4	
Water Intake	Believes drinking a lot of water affects PKD	10	Told to drink “a lot of water”	12	Adheres to recommendations	10	• Frequent urination • Experiences symptoms • Difficult to develop habit
			• By non-physician sources	3	Does not adhere to recommendations	1	
	Uncertain	5			Uncertain but tries to adhere to recommendations	5	

Similarly, many of the 14 participants who reported believing exercise and physical activity affects PKD (Table 2) maintained specific regimens. For example, several participants reported walking at least 45 min each day. One participant’s health philosophy was to “use (his body) or lose it,” so he walked for 45 min about twice per week, swam for 30 min every other day, and also did exercises prescribed by his chiropractor. Another participant went to a fitness club to use a stationary bicycle three times per week and work with a trainer once a week because “people who are fit…probably do better than people who lay around on the couch.”

When asked about adherence to low-salt diet, most believed low-salt diets were of benefit. Fourteen participants mentioned they do not add salt to food, four said they avoid salty food, and four did not eat out at restaurants and/or cooked their own meals at home (Table 2). For one individual, maintaining a low salt diet was “life or death.” She had been placed on the transplant list and was told by a doctor that she would be on dialysis within six months. However, she stated, “I lowered my (creatinine) and my BUN and all that, just by diet…and here we are a little over a year (and I’m) still not on dialysis.” Participants worked to maintain these diets despite challenges, including interference with the social aspect of eating and difficulty measuring the amount of nutrients consumed. One participant recalled an incident when she felt ill after a family event because she ate food that she was advised to avoid “rather than insult them (by refusing).” She explained that she would now “do it differently…because you’re going to pay the price with your body.” Another participant had difficulty maintaining her diet when with friends or traveling because she would eat out at restaurants more frequently. To avoid this challenge, she started bringing her own snacks because her thought was “do you want to eat this, or do you want to go on dialysis?”

Ten participants reported believing that drinking a lot of water affects PKD, while 5 were uncertain about its effects (Table 2). Twelve participants received recommendations to drink “a lot of water,” with 3 out of 12 receiving them from non-physician sources, including family members and PKD support groups. Ten participants reported that they adhered to the recommendations, while five were uncertain about their adherence but tried to follow the recommendations (Table 2). The majority of participants did not keep track of their water intake, but they incorporated strategies, such as using a bottle or pitcher that they kept filled, to increase water intake. One participant reported trying to drink three 33-oz bottles of water per day because “it eliminates a lot of the pain for me” despite the fact that “it’s challenging because you have to pee every 20 minutes.” Another individual recalled how after starting on a diet that required him to drink a lot of water, he had to be hospitalized because his “salt level was too low and that I needed to stop drinking so much water.”

### Theme 2: Personal beliefs contrasting with treatment recommendations

Patient beliefs about the efficacy and appropriateness of various treatments affected their self-care behavior. For the four participants who reported taking medication fewer than 7 days per week, one challenge with adhering to the medication regimen was the belief that diet and exercise were more effective than medications (Table 2). One participant had taken blood pressure medication for over 20 years until he started seeing a chiropractor, who focused him on changes in diet and exercise. He explained, “I had some hypertension, and now I don’t have hypertension. Now I changed my diet and changed my exercise, so somehow I perceive that’s better than the drugs.”

Some patients became motivated to take their medication when they believed, from personal experience, reading, or being told by others, that medication is important for blood pressure control. For example, one participant realized “I needed to stick to the medication” because “even though I was really trying to do everything (in terms of exercise and diet) to keep my blood pressure down, there was really nothing I could do (to lower blood pressure).” In another case, the participant explained, “I was told I have to take my blood pressure medication and all that, I thought, I really don’t. My dad [who has PKD] is fine…When I started educating myself…is when I went…“You should at least take the lisinopril because you know your blood pressure is high.” Similarly, another participant initially believed that she could lower her blood pressure with exercise. She became more willing to take her blood pressure medication when a doctor “had mentioned...that I don’t have (the type of) kidney disease where your blood pressure can be lowered because of diet or exercise.”

Eleven participants had been told to avoid certain types of exercise or physical activity, including contact sports and weight lifting. Two participants reported deliberately going against these recommendations because the activity helped them psychologically and they had not experienced any adverse effects (Table 2). One participant’s doctor told her to avoid weight lifting, but she continued to do so because “it helps me mentally” and “I don't have any side effects from it at all.” She further explained, “[My doctor] just thinks I’m too extreme…I feel like it’s my sanity. Until it makes a difference for me, I don’t see it as a problem.” Similarly, despite being told to not lift anything over 25 lb, another participant admitted, “I do probably lift sometimes 40 pounds, but I don’t do anything that I feel I’m straining myself…I think the healthier you are and more fit you are, that you’re going to probably feel better.”

### Theme 3: Confusion due to unclear recommendations

Although fifteen participants had been told to follow certain diets, only 5 out of 15 received these recommendations from their physician. The remainder learned of dietary restrictions from nutritionists, dieticians, family members, and articles (Table 2). One individual knew to maintain a low-salt diet because her mother, who also had PKD, never added salt to her food. However, her physicians never told her to follow a certain diet until “towards the end or closer to my transplant” when “it took me prompting (my nephrologist) to recommend (a referral to a dietician).” In retrospect, she now believed “a dietician maybe would be a good thing” because it would have been really helpful to know “sodium levels in food…what I could look for.” Another participant expressed a desire to know more from her physician about “dietary recommendations and lifestyle changes that could help her kidneys, instead of just ordering labs and saying, ‘Your labs look good. See you next year.’”

Many participants also reported receiving confusing or conflicting dietary recommendations. One participant recalled that her nutritionist would recommend “a portion,” which she found unhelpful because “what is a portion?” Consequently, she had done reading and used the internet for dietary recommendations. To add to the confusion, one participant recalled being referred to a program for kidney disease patients that included classes on how to eat. However, she later learned at PKD meetings that “diet care just for kidney failure is different than kidney failure from PKD” and there are “a lot of different things that (PKD patients) are not supposed to have.”

Another participant summarized the challenge of having uncertain recommendations: “The hardest part is because you don’t know that any of it is actually beneficial. So part of you could just be like, screw it, I’ll just drink coffee everyday and have steak and hamburgers for dinner…Or I could be like, well it might be making my kidneys worse, so I’m going to limit everything and suffer a little bit now in hopes that maybe I’ll prolong the demise from this disease.”

Several participants received conflicting information about water intake. One participant recalled, “I’ve been told to drink a lot of water or you can drink too much water and it’s going to be a bad thing for you.” She explained that her doctor always told her to stay hydrated but “doesn’t tell me what to drink.” On the other hand, she had heard at research meetings and support groups that “there’s been studies that show if you drink this much water that it will help keep you function at a steady pace.” Because “they just contradict themselves,” she “just drinks the recommended dose for any given person.”

Similarly, another participant had read a study about drinking six to eight liters of water per day instead of two to three liters per day. When she mentioned this to her first nephrologist, she was told, “It does sound like a good idea.” When she switched nephrologists, the new one told her, “That study was inconclusive…drink your two liters of water a day and don’t worry about drinking any more.” As a result, the participant reported feeling “kind of left just blank like I don’t know.” Another individual reported that he “read there’s no evidence that (drinking a lot of water is beneficial).” However, his nephrologist “believes that it’s the right way to go.”

## Discussion

We found that PKD patients are motivated and willing to incorporate blood pressure control, exercise, low-salt diet, and high volume water intake into their daily routines. Seventy-five percent of the participants who were prescribed blood pressure medication reported adherence, which is higher than the reported 50% medication adherence rates for chronic diseases [15, 16]. Similarly, a majority of the participants reported adhering to exercise, low-salt diet, and high volume water intake recommendations. This is in contrast to generally low adherence rates for lifestyle changes in hypertension management, with less than 10% long-term success rates for weight loss and smoking cessation [15] as well as low rates of achieving desired salt reduction in clinical trials [17].

The higher adherence rates in this study may be due to the patients’ beliefs that medication and lifestyle changes are necessary for slowing the progression of their disease, as suggested by a patient who believed diet lowered her creatinine levels and another patient who became more willing to take her blood pressure medications after lifestyle changes failed to lower her blood pressure. This is consistent with the Ross et al. study describing patients’ beliefs and compliance, which found a strong correlation between compliance and belief in the necessity of the intervention [13].

However, personal beliefs can also be a barrier to adherence. Ross et al. also found that individuals who believed they could control their blood pressure with lifestyle changes were less likely to be adherent with their medications [13]. Similarly, the patients in the study who did not adhere to recommendations reported personal experiences that went against the recommendation. For example, one patient in our study did not take his blood pressure medication as prescribed because he believed that exercise and diet were more effective for blood pressure control.

Another barrier to adherence that we identified was confusion due to unclear recommendations, especially about water intake. Many participants reported receiving conflicting or confusing instructions from physicians or only receiving recommendations from non-physician sources. This is consistent with the Bell and Kravitz study showing that physicians mention the importance of physical activity/exercise, healthy eating, and sodium restriction in only 54.2%, 38.3%, and 14.2% of office visits for hypertension, respectively [20]. This may also be due to current lack of evidence for best practice for increasing water intake in various kidney-related diseases including but not limited to PKD [21]. In a systematic review of 16 studies investigating high fluid intake and chronic kidney disease including PKD, recurrent nephrolithiasis, and urinary tract infection, interventions ranged from increasing water intake by 1.5 to 3 l. [21] In the PKD population, although this may be due to different targets for water intake recommended at different stages of kidney disease, most patients in our study did not indicate that they received varying instructions based on disease stage.

These findings suggest there is variability between what patients understand about their disease and prescribed treatments and what they believe their doctors have told them. First, not all physicians focus on lifestyle-based treatments, but the majority of PKD patients in our study reported desiring specific recommendations on how to implement these and sought information from non-physician sources. Many patients expressed a desire for greater engagement from their nephrologists in addressing modifiable lifestyle choices as well as greater communication. This echoes the desire for greater physician contact during the “latent period” between diagnosis and symptom manifestations observed by Baker et al. in a previous study in England [3]. This also suggests that a multidisciplinary approach including dietitians, primary doctors, and other specialists may be beneficial for PKD patients.

Second, participants experienced confusion about recommendations, which further supports a need for improving communication between physicians and patients. While patients may be receiving varying advice due to limited evidence or different stages of kidney disease, these should be made clear to patients. Furthermore, it may be helpful to provide more specific instructions given that participants expressed confusion over vague recommendations. Studies support that providing specific instructions including the amount and frequency of water intake (i.e. drink 500 mL of water three times daily before meals) achieved more consistent changes [21].

This study has strengths and limitations. Our interview strategy allowed participants to freely express their personal beliefs, whether or not these agreed with physician recommendations. We used an interview guide but provided opportunities for participants to discuss their responses in an open-ended fashion. All interviews were conducted by a single interviewer who was not known to the patients prior to the interviews, allowing for impartiality. The strength of the qualitative method in this instance is to learn, from the patient’s perspective, how beliefs and practices regarding PKD care develop, given that there are not widely recognized or disseminated guidelines for how patients should take care of their disease. Qualitative inquiry is particularly suited to situations of ambiguity. Because guidelines for self-care in PKD are ambiguous, and can change over the course of the disease, patients need to interpret and adapt what they have heard regarding self-care. The qualitative approach employed is sensitive to variations in patient beliefs and efforts at self-care. Limitations of the study included the small number of patients represented, and the fact that all were from the Northern California region. However, the participants were drawn from many different nephrology practices and qualitative research conducted with a rigorous approach generally allows for a smaller sample size than quantitative research studies. In addition, the participants voluntarily responded to a survey sent to the national PKD foundation email list, which may be selecting for individuals who are more proactive and motivated to pursue interventions. Those who are members of the email list may also be more technologically savvy and/or of higher socioeconomic status with access to a computer and the Internet, allowing for greater exposure to information and resources. Although this may limit generalizability, the themes that emerged from these interviews represent important areas that warrant further evaluation in the broader PKD community. We recognize that a deeper exploration in a larger sample may reveal other themes and sub-themes. The current study also did not allow for obtaining physician perspectives.

## Conclusions

Overall, most PKD patients in our study were highly motivated to pursue therapies they believed to be helpful whether prescribed by a doctor or another source of information. These findings support a role for further exploring patient beliefs about the disease and its necessary treatments in order to design strategies to improve communication and meet the needs of these patients. Participants also experienced some confusion about topics including lifestyle change, which suggests the need for clarity and detail especially when prescribing lifestyle modifications.

## Additional file

Additional file 1: Interview Guide: List of questions used to guide patient interviews (DOCX 18 kb)

## Abbreviations

ACE-I: Angiotensin converting enzyme inhibitor
ADH: Anti-diuretic hormone
BUN: Blood urea nitrogen
HALT-PKD: Halt progression of polycystic kidney disease
IRB: Institutional review board
PKD: Polycystic kidney disease
UCSF: University of California, San Francisco
