# Supplementary material for: Understanding barriers to medication, dietary, and lifestyle treatments prescribed in polycystic kidney disease
Source: BMC Nephrol. 2017 Jul 5;18:214. doi: 10.1186/s12882-017-0641-3 (PMC5498983; doi:10.1186/s12882-017-0641-3)
Supplement: Additional file 1: — Interview Guide: List of questions used to guide patient interviews (DOCX 18 kb) [file 12882_2017_641_MOESM1_ESM.docx]

Interview #1 ILLNESS BELIEFS AND PRACTICES – Polycystic Kidney Disease

Introductions

Review the goals of the interview

(Informed consents will already be obtained at clinic visit.)

Review the plan to audiotape the interview.

INTERVIEW QUESTIONS Patient version of interview questions.

(Questions in bold guide the interview. Probes are asked if needed to fill out the informants’ answers.)

1. Can you tell me how you understand your polycystic kidney disease? What has made it stay the same or get worse over time?

2. What kind of treatment do you think is necessary for PKD?

Probes:

Have you ever been told there is no treatment?

What do you think about the treatment that Western doctors and clinics offer?

Consider Medications, recommendations for diet, exercise.

Consider herbs, medicines, acupuncture, alternative treatments.

3. What are the most important results that you hope you will receive from these treatments?

4. What do you think the future brings for your PKD?

Probes:

What do you fear most about PKD in the future?

What do you hope for most for PKD in the future?

5. In your everyday life, for example, yesterday, how were you aware of your illness?

Probes:

What problems did the illness cause you?

What problems did the illness cause your family?

Now I’d like to switch a bit and learn about what you have been told about how to care for your illness, and how you have used that information in managing your illness.

Part I. Blood pressure

1. Do you take an angiotensin converting enzyme inhibitor (ACEI) or angiotensin II receptor blocker (ARB)?

Probes:

Examples of ACEi – lisinopril, captopril, benazepril

Examples of ARB - losartan

1. Approximately how many days of the week do you remember to take your medication?
2. What makes a difference in whether you remember?

Probes:

Stress, vacation, feeling well/unwell, etc.?

Part II. Physical activity / exercise.

1. What kind of exercise or physical activity do you enjoy?

2. What kind of exercise or physical activity do you do on a daily basis?

3. Has anyone ever told you to avoid certain types of exercise or physical activity?

4. Do you experience any of the following (and indicate how many days per week)

Back pain

Abdominal pain

Side pain

Leg pain

Leg cramps

Headaches

Chest pain

5. Have you noted any changes in your symptoms with certain types of exercise or physical activity?

Probes:

Symptoms get better or worse?

Weight loss? Appetite changes? Thirst changes?

6. Has your level of activity changed since you were diagnosed with PKD? If so, how?

Part III. Low-salt diet.

1. Have you been told to follow a low-salt diet?
2. To what extent do you believe you follow a low-salt diet?
3. How do you keep track of how much salt you consume per day?
4. Please give an approximation (in any type of unit- mg, cups, teaspoons) of how much salt you consume in one day.

Part IV. High water volume intake.

1. Have you been told to drink “a lot of water”?

2. Have you ever received specific guidelines on how many glasses of water to drink? If so, what were these guidelines?

3. How much do you think this means to you?

4. How do you understand that drinking a lot of water might affect your disease?

5. How do you keep track of how many glasses of water you drink per day?

6 . How many glasses of water do YOU think you drink on average per day?

Thank you for participating in this interview.
